# Combination immunotherapy and radiotherapy causes an abscopal treatment response in a mouse model of castration resistant prostate cancer

**DOI:** 10.1186/s40425-019-0704-z

**Published:** 2019-08-14

**Authors:** Stephanie O. Dudzinski, Brent D. Cameron, Jian Wang, Jeffrey C. Rathmell, Todd D. Giorgio, Austin N. Kirschner

**Affiliations:** 1 Vanderbilt University School of Medicine, Nashville, TN 37232 USA; 2 Department of Biomedical Engineering, Vanderbilt University, Nashville, TN 37232 USA; 3 Department of Radiation Oncology, Vanderbilt University Medical Center, B1003 PRB, 2220 Pierce Avenue, Nashville, TN 37232 USA; 4 Department of Pathology, Microbiology and Immunology, Vanderbilt University Medical Center, Nashville, TN 37232 USA; 5 Vanderbilt Center for Immunobiology, Vanderbilt University School of Medicine, Nashville, TN 37232 USA

**Keywords:** Immunotherapy/Antibody immunotherapy, Radiation oncology/combined modality therapies, Model organisms/animal models of cancer, Genitourinary cancers/prostate cancer, Radiobiology/tumor microenvironment and modification, Abscopal effect, Castration-resistant prostate cancer, Immuno-radiotherapy

## Abstract

**Background:**

Prostate cancer is poorly responsive to immune checkpoint inhibition, yet a combination with radiotherapy may enhance the immune response. In this study, we combined radiotherapy with immune checkpoint inhibition (iRT) in a castration-resistant prostate cancer (CRPC) preclinical model.

**Methods:**

Two Myc-CaP tumor grafts were established in each castrated FVB mouse. Anti-PD-1 or anti-PD-L1 antibodies were given and one graft was irradiated 20 Gy in 2 fractions.

**Results:**

In CRPC, a significant increase in survival was found for radiation treatment combined with either anti-PD-1 or anti-PD-L1 compared to monotherapy. The median survival for anti-PD-L1 alone was 13 days compared to 30 days for iRT (*p* = 0.0003), and for anti-PD-1 alone was 21 days compared to 36 days for iRT (*p* = 0.0009). Additional treatment with anti-CD8 antibody blocked the survival effect. An abscopal treatment effect was observed for iRT in which the unirradiated graft responded similarly to the irradiated graft in the same mouse. At 21 days, the mean graft volume for anti-PD-1 alone was 2094 mm^3^ compared to iRT irradiated grafts 726 mm^3^ (*p* = 0.04) and unirradiated grafts 343 mm^3^ (*p* = 0.0066). At 17 days, the mean graft volume for anti-PD-L1 alone was 1754 mm^3^ compared to iRT irradiated grafts 284 mm^3^ (*p* = 0.04) and unirradiated grafts 556 mm^3^ (*p* = 0.21). Flow cytometry and immunohistochemistry identified CD8+ immune cell populations altered by combination treatment in grafts harvested at the peak effect of immunotherapy, 2–3 weeks after starting treatment.

**Conclusions:**

These data provide preclinical evidence for the use of iRT targeting PD-1 and PD-L1 in the treatment of CRPC. Immune checkpoint inhibition combined with radiotherapy treats CPRC with significant increases in median survival compared to drug alone: 70% longer for anti-PD-1 and 130% for anti-PD-L1, and with an abscopal treatment effect.

**Precis:**

Castration-resistant prostate cancer in a wild-type mouse model is successfully treated by X-ray radiotherapy combined with PD-1 or PD-L1 immune checkpoint inhibition, demonstrating significantly increased median overall survival and robust local and abscopal treatment responses, in part mediated by CD8 T-cells.

**Electronic supplementary material:**

The online version of this article (10.1186/s40425-019-0704-z) contains supplementary material, which is available to authorized users.

## Introduction

Prostate cancer is the most common non-cutaneous malignancy and the second-leading cause of cancer-related mortality among men in the USA, with distant disease having a 5-year survival rate of 29.8% [1]. Though metastatic disease is initially responsive to androgen blockade, over time this treatment selects for a castration-resistant prostate cancer (CRPC) population with modern systemic treatments providing a median survival of 2.8 years, albeit with significant quality of life detriment due to treatment-related effects and disease progression [2].

The combination of immunotherapy and radiotherapy is an emerging clinical treatment paradigm, a growing research sector, and a critical research domain supported by the Radiation Biology Task Force [3]. X-ray radiation treatment (XRT) can activate both the adaptive and innate immune systems through directly killing tumor cells, causing mutations in tumor-derived peptides, and causing localized inflammation that increases immune cell trafficking to tumors [4, 5]. Most importantly, the activated immune system may cause tumor-directed treatment responses away from the site of irradiation, i.e., an abscopal treatment effect, which has the potential to treat disease throughout the body.

However, prostate tumors are considered poorly responsive to immunotherapy due to their low genetic mutational load, their lack of activated tumor-infiltrating lymphocytes, and specific genetic alterations that influence the immune landscape [6, 7]. Studies have shown over 50% of aggressive prostate cancers express high levels of PD-L1, a key factor in suppressing the local immune response [8]. A negative regulator of the immune response, Tregs have also been found to be enriched in both the tumor and peripheral blood of patients with prostate cancer [9, 10]. Altogether, prostate cancer has mechanisms to evade and inhibit anti-tumor immunity.

Clinical trials have studied immune checkpoint inhibition for CRPC. One phase III randomized trial of 799 patients tested 8 Gy XRT to a CRPC bone metastasis followed by either placebo or 4 cycles of ipilimumab (anti-CTLA4) and found the combination provided a statistically significant 7 mo median survival increase in a post-hoc analysis of a predefined subgroup of patients with low tumor burden (22.7 mo vs 15.8 mo, *p* = 0.0038) [11]. However, only a trend for improved overall survival was seen for the whole cohort (11.2 mo vs 10.0 mo, *p* = 0.053) and therefore the trial did not provide enough evidence to meet its primary endpoint and influence clinical practice [11]. A subsequent phase III randomized clinical trial for 600 patients with low tumor burden tested ipilimumab versus placebo, without XRT [12]. However, this ipilimumab-only approach failed to show any overall survival benefit and only a marginal progression-free survival benefit and prostate-specific antigen (PSA) response was seen, suggesting that the combination with XRT produces a superior treatment response in patients with low disease burden [12]. These large randomized clinical trials indicate there is a potentially powerful treatment approach when combining radiotherapy with immunotherapy for CPRC, but the optimal treatment combination has not yet been found for most patients to derive benefit.

This project builds upon the findings of these clinical trials to develop preclinical models that can be used to optimize the treatment approach. Anti-PD-1 and anti-PD-L1 antibodies are immune checkpoint inhibitors that target tumor-immune cell interactions and clinically have a reasonably favorable side-effect profile in patients. This suggests PD-inhibitors may be superior to anti-CLTA4 agents, which primarily block the interaction between immune cells without directly involving the tumor. However, PD-1 agents alone show little response in treating CRPC in early phase clinical trials [13]. Nevertheless, logically following the clinical trials described above, we hypothesized that combination PD-based immunotherapy-radiotherapy (iRT) approach would trigger a robust treatment response against CRPC that is mediated through the immune system, causing both local and distant (abscopal) effects, while likely being better tolerated in patients than an anti-CTLA4 approach.

There is evidence to suggest that the tumor-dependence on PD-1/PD-L1 immunosuppression is enhanced in lesions that respond to radiation [14]. Therefore, we examine a combination of immune checkpoint inhibition and radiotherapy for CRPC that causes local and abscopal treatment effects mediated by activated immune cells.

## Materials & methods

### Cell lines

Myc-CaP cells were purchased from ATCC, authenticated by short tandem repeat analysis and confirmed *Mycoplasma*-free (CellCheck Mouse Plus, IDEXX BioAnalytics, Columbia, MO), and grown in cell culture in DMEM medium (Corning) supplemented with 10% fetal bovine serum (Corning) and 1% penicillin-streptomycin (Gibco).

### Mouse model of immuno-radiotherapy

A mouse prostate cancer model that mimics common human CRPC was developed. Myc-CaP tumors were engrafted into FVB mice (JAX) from which the tumor cells were derived [15]. Injecting one million cells in 50–70% Matrigel (Corning), two subcutaneous tumors were simultaneously established in each mouse, one in the flank and one in the hindlimb (leg). After the tumor grafts reached 500 mm^3^, castration was performed, and after brief regression the tumors continued to grow castration-resistant [16]. Mice were then treated with either anti-mouse PD-1 (clone RMP1–14, Bio X Cell) or PD-L1 (clone B7-H1, Bio X Cell) antibody, 0.2 mg IP given on days 0, 2, 4, and 7. Only leg tumors were treated with XRT 20 Gy in 2 equal treatments given on days 7 and 8. Survival was assessed as the primary outcome. A separate cohort was treated similarly and tumors were harvested on days 14–17 for flow cytometry and immunohistochemistry analyses. Treatment cohorts were repeated at least 3 times with adequately powered numbers of mice per group with similar results. Representative data from example cohorts are presented in the figures.

The CD8-depleted mouse cohort was treated as above and given anti-mouse CD8a antibody (clone 2.43, Bio X cell), 0.2 mg IP given on days 7, 14, and 21. All research involving vertebrate animals was performed in strict accordance with protocols M/14/182 and M1700134 approved by Vanderbilt’s Institutional Animal Care and Use Committee (IACUC). All procedures were conducted according to applicable national guidelines, including appropriate analgesics and anesthesia to ameliorate and minimize animal suffering.

### Immunohistochemistry

Harvested tumor grafts were fixed in 10% zinc-formalin (Fisher Scientific) at room temperature overnight, then transferred to 70% ethanol for paraffin embedding. Immunohistochemical staining for Ki67 and cleaved caspase-3 was performed on serial sections. Whole slide digital imaging was analyzed using QuPath software for positive cell counts, using sigma level 2.0 and threshold level 0.3 [17].

### Tumor dissociation

To analyze the tumor immune microenvironment during the anticipated efficacious period of immune checkpoint activity, tumors were collected at day 14–17 after starting immune checkpoint inhibitor. Fresh tumors were dissociated into single cell suspensions with DNAse I (Invitrogen), collagenase Type IV (Sigma), and hyaluronidase (MP Biomedicals) for 1 h at room temperature using a dissociator (Miltenyi) with gentleMACS C-tubes. To remove calcium, cells were resuspended for 5 min in HBSS without calcium or magnesium (Gibco), then resuspended in 5 mM of EDTA for 30 min at room temperature. Next, cells were passed through a 70 μm filter before ACK lysing buffer (KD Medical Inc) was added to remove red blood cells before flow cytometry. Immediate staining was performed for surface marker expression to analyze with flow cytometry.

### Fluorescence cytometry

One million cells of each tumor were transferred to a 96-well round-bottom, micro test plate and pelletized at 1500 rpm for 5 min (Beckman-Coulture Allegra X-14 Centrifuge). A fixable viability dye (eBioscience, eFluor 780) was used to identify live cells. The following antibodies were used for surface staining: CD3 APC (Biolegend, Clone: 17A2), CD4 BV510 (BD Bioscience Clone RM4–5), CD8a eFluor 450 (eBioscience, Clone: 53–6.7), CD279 (PD-1) FITC (eBioscience, Clone: J43), CD44 PECy5 (eBioscience, Clone: IM7), CD335 PECy7 (Biolegend, Clone: 29A1.4), CD11b AF488 (Biolegend M1/70), F4/80 BV421 (Biolegend BM8), CD206 PE (Biolegend C068C2), CD86 APC (Biolegend GL-1). Briefly, cells were stained with Fc blocking antibodies (TruStain FxX Biologend) for 10 min at 4 °C followed by cell surface antibodies in FACS Buffer (PBS with 2% FBS) for 30 min at 4 °C. Cells were pelletized at 1500 rpm for 5 min before re-suspending in 200 μL of FACS Buffer. Expression of T cell surface markers was measured by fluorescence cytometry (MACSQuant, Miltenyi Biotec) and analyzed by FlowJo software (Tree Star Inc.).

### Statistical methods

Graft volumes were compared at the indicated timepoint using a one-tailed T-Test for two-samples with unequal variance (Microsoft Excel). Survival was compared using log-rank (Mantel-Cox) test (GraphPad Prism). Immunohistochemical staining was analyzed by one-way ANOVA with Tukey’s test for multiple comparisons, where *p*-values of < 0.05 were considered statistically significant (GraphPad Prism). Flow cytometry comparisons of Control, Flank, and Leg tumors were analyzed using a two-way ANOVA, where *p*-values of < 0.05 were considered statistically significant (GraphPad Prism).

## Results

**Fig. 1:**
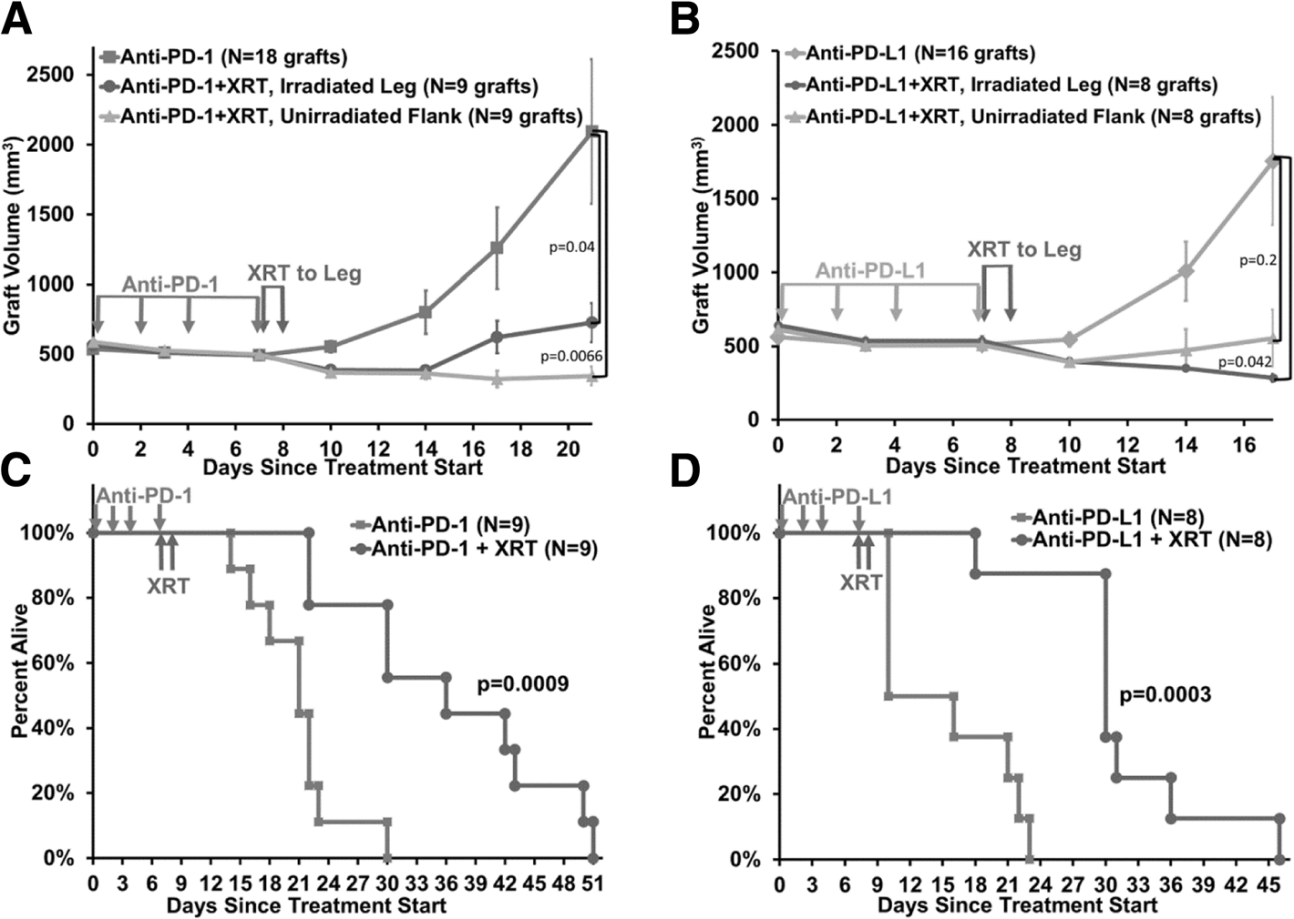
Castration-resistant prostate cancer is successfully treated by immune checkpoint inhibitor combined with radiotherapy, with effects on the irradiated and unirradiated tumors, and increased survival. **a-d.** Myc-CaP tumor graft volumes (**a and b**) and survival (**c and d**) for mice treated with immune checkpoint inhibitor monotherapy and given in combination with XRT to the leg graft. Significantly decreased tumor graft volume and significantly increased median survival was observed. Error bars represent ± SEM

We developed a PD-based iRT approach for CRPC in an immunocompetent castrated syngeneic FVB mouse model using subcutaneous Myc-CaP tumor grafts [16, 18]. Expression of PD-L1 in Myc-CaP cells increases after irradiation (Additional file 1: Figure S1). Compared to mice treated with antibody alone, XRT (20 Gy in 2 fractions) to the leg tumor graft causes a local response in the irradiated tumor and a robust abscopal effect with regression of an unirradiated distant tumor graft (Fig. 1a and b). At 21 days, the mean graft volume for anti-PD-1 alone was 2094 mm^3^ (*N* = 18 grafts) compared to iRT irradiated grafts 726 mm^3^ (*N* = 9 grafts) (*p* = 0.04) and unirradiated grafts 343 mm^3^ (*N* = 9 grafts) (*p* = 0.0066). At 17 days, the mean graft volume for anti-PD-L1 alone was 1754 mm^3^ (*N* = 16 grafts) compared to iRT irradiated grafts 284 mm^3^ (*N* = 8 grafts) (*p* = 0.04) and unirradiated grafts 556 mm^3^ (*N* = 8 grafts) (*p* = 0.21). No significant differences were observed between the leg and flank graft volumes within each treatment group, so both grafts were included in the antibody alone data. Additional tumor graft volume data is in Additional file 1: Figure S2.

Remarkably, this combined iRT approach significantly increased survival (Fig. 1c and d). For anti-PD-L1, the median survival for antibody alone was 13 days (*N* = 8 mice) compared to 30 days (*N* = 8 mice) for iRT (*p* = 0.0003). For anti-PD-1, the median survival for antibody alone was 21 days (*N* = 9 mice) compared to 36 days (*N* = 9 mice) for iRT (*p* = 0.0009).

**Fig. 2:**
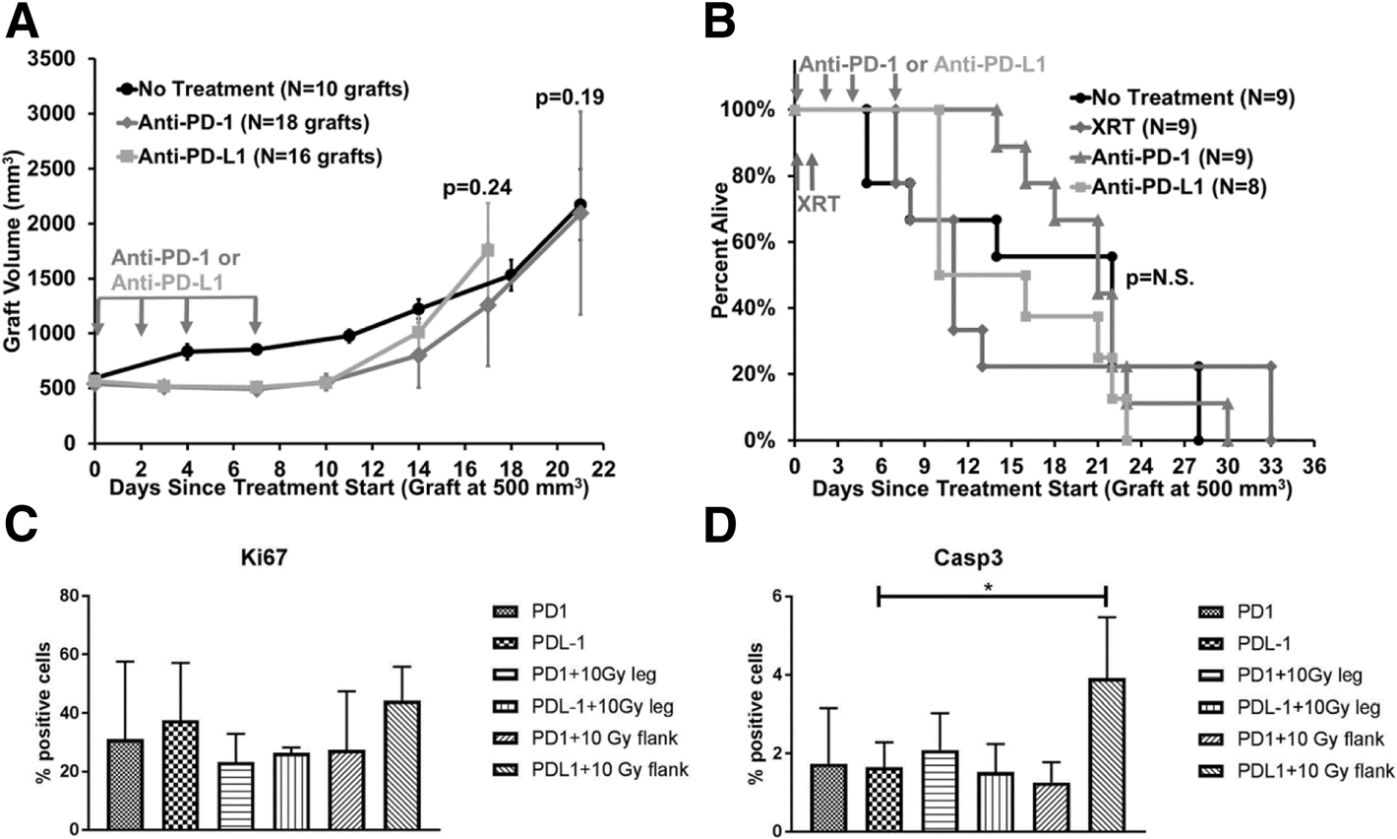
Monotherapy is similar to no treatment, and tumor cell proliferation and apoptosis is minimally effected. **a.** Myc-CaP tumor graft volumes for untreated mice and those treated with immune checkpoint inhibitor monotherapy. **b.** Survival of mice with Myc-CaP grafts, including no treatment, XRT alone, and immune checkpoint inhibitor monotherapy, as indicated, with no significant differences in median overall survival between the groups (log-rank test, *p* > 0.05). **c and d.** Ki67 and cleaved caspase-3 (Casp3) immunohistochemical staining analyzed by whole slide digital imaging. Independent graft numbers analyzed: anti-PD-1 (*N* = 4), anti-PD-L1 (*N* = 6), anti-PD-1 + XRT leg graft (*N* = 4), anti-PD-L1 + XRT leg graft (*N* = 2), anti-PD-1 + XRT flank unirradiated graft (*N* = 3), anti-PD-L1 + XRT flank unirradiated graft (*N* = 3). Error bars represent ± SEM

Similar to clinical data showing lack of efficacy for immune checkpoint monotherapy, we found that mean graft volume for untreated grafts was similar to anti-PD-1 (*p* = 0.19) and anti-PD-L1 (*p* = 0.24) antibody treatment alone, respectively (Fig. 2a). Furthermore, the survival of mice without treatment or those treated with XRT alone were similar (*p* = N.S.) to those treated with anti-PD-1 alone or anti-PD-L1 alone (Fig. 2b). This indicates the importance of combination treatment over monotherapy in this preclinical model.

To study treatment-related effects on tumor cell proliferation and apoptosis, immunohistochemical staining was performed on grafts harvested at day 16 after starting immune checkpoint inhibitor treatment. There were no differences among the treatment groups for Ki67 or cleaved caspase-3 staining, except for a significant increase in caspase staining (*p* = 0.024) in the unirradiated flank tumor when analyzing anti-PD-L1 combined with XRT compared to anti-PD-L1 monotherapy (Fig. 2c and d). The mechanism for this finding is under investigation.

**Fig. 3:**
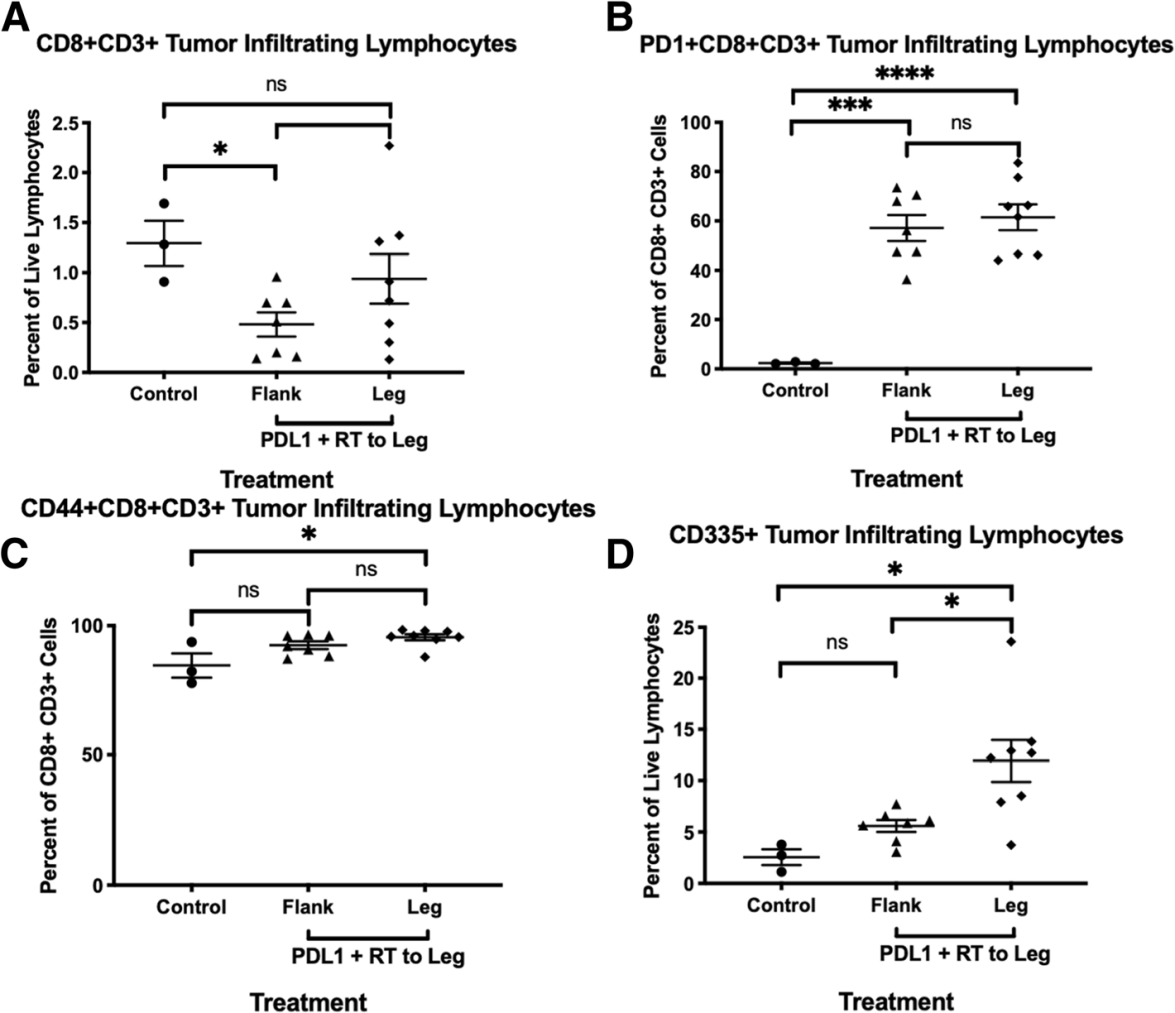
Immune profile in tumor microenvironment. **a-c.** Flow cytometry for double positive CD8+ and CD3+ T cells and expression of selected markers (PD-1 or CD44) on CD8+ T cells within Myc-CaP untreated control tumors (*N* = 3), flank tumors from mice treated with anti-PD-L1 and XRT to leg tumor (*N* = 7), or leg tumors that received direct XRT and ant-PD-L1 treatment (*N* = 8). **d.** Flow cytometry for CD335+ NK cells from live cells within Myc-CaP untreated control tumors (*N* = 3), flank tumors from mice treated with anti-PD-L1 and XRT to its leg tumor (*N* = 7), or leg tumor that received direct XRT and anti-PD-L1 treatment (*N* = 8). Error bars represent ± SEM; *P < 0.05, **P < 0.01, and ***P < 0.001, and ****P < 0.0001, two-way ANOVA test

Based on other iRT tumor models, we hypothesized that an immune-related treatment mechanism may be mediated by tumor-infiltrating immune cells, especially CD8+ T cells. The tumor microenvironment was studied by flow cytometry on tumor tissue harvested at day 14–17 after starting immunotherapy, which provides quantification of tumor infiltrating lymphocytes (TILs). After selecting live lymphocytes, appropriate T cells populations were selected using double positive CD8 + CD3+ gates or CD4 + CD3+ gates, while natural killer (NK) cells were selected as live lymphocytes that are CD335+. There was a greater percent of CD8 + CD3+ cytotoxic T cells in the untreated control tumors compared to those treated with anti-PD-L1 and XRT (Fig. 3a).

T cells were then analyzed for markers of exhaustion and activation. In mice treated with anti-PD-L1 antibody and XRT to the leg tumor, both flank and leg tumors had significantly higher expression of PD-1 in CD8 + CD3+ cytotoxic T cells (Fig. 3b). Additionally, the leg tumor treated with radiation and anti-PD-L1 had higher CD44+ expression on CD8 + CD3+ cytotoxic T cells compared to untreated control tumors. (Fig. 3c). Furthermore, CD335+ tumor infiltrating CD335+ cells were significantly increased in the grafts treated with anti-PD-L1 and XRT compared to untreated control (Fig. 3d).

**Fig. 4:**
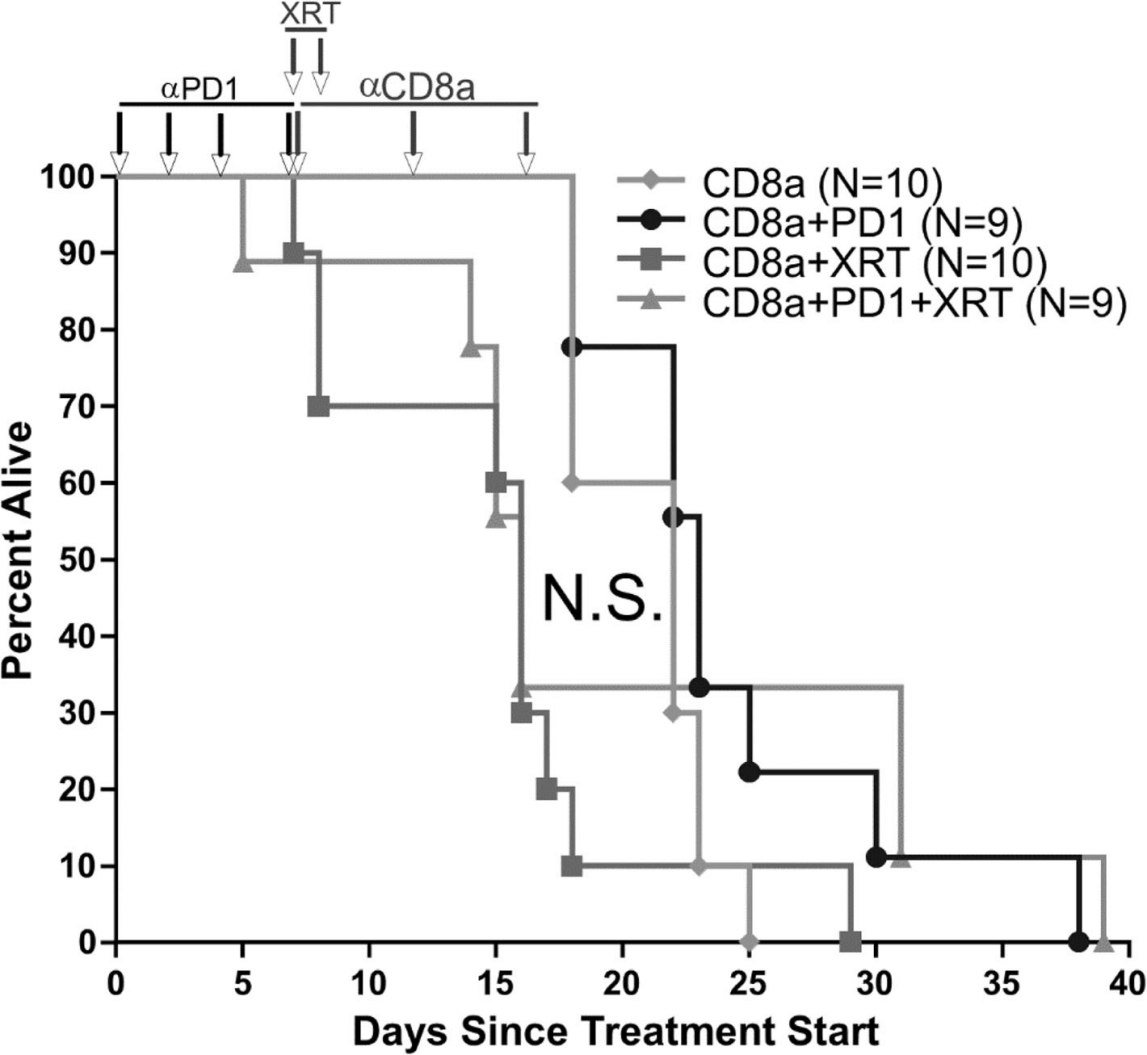
CD8 depletion blocks survival advantage from combination radiotherapy and immune checkpoint inhibition. Survival for mice with Myc-CaP tumor grafts treated with anti-CD8a antibody to deplete CD8+ cells. Mice received no additional treatment, anti-PD-1 immune checkpoint inhibitor alone, XRT alone, or anti-PD-1 in combination with XRT to the leg graft. N.S. = non-significant median survival difference

The role of CD8+ cells in iRT response was verified in the Myc-CaP CRPC mouse model by depleting CD8+ cells by three once-weekly injections of anti-CD8a antibody [19]. The results show loss of the survival advantage, which suggests that part of this iRT mechanism is mediated by a CD8+ cell (Fig. 4a). Additional flow cytometry data and gating strategy is in Additional file 1: Figure S3 and S4.

## Discussion

Although clinical data suggests limited effects of immune checkpoint inhibitor treatment for CRPC, this preclinical model indicates robust responses are achievable using when combining anti-PD-1 or anti-PD-L1 treatment with XRT. A syngeneic mouse model was selected to allow treatment effects to be studied in the presence of an intact immune system. The highly-aggressive Myc-CaP model in the castration-resistant setting was selected to investigate treatment efficacy.

Tumor graft growth was significantly diminished by the combination treatment of immune checkpoint inhibitor and XRT compared to drug alone. Remarkably, unirradiated distant tumor grafts also responded to combination treatment, suggesting an abscopal treatment effect. Most importantly, significant increases in median survival were observed compared to antibody treatment alone: 70% longer for anti-PD-1 and 130% for anti-PD-L1. Importantly, no increased toxicity was observed for combination immuno-radiotherapy treatment compared to monotherapy. However, a notable limitation of this preclinical model is that the combination treatment was not found to be durable after a single treatment cycle (8 days), with no mice completely clearing their tumor grafts. It is possible that repeat dosing by immune checkpoint inhibitor would extend the treatment effect, as found in clinical studies using immune checkpoint inhibitors, but this was not investigated in this preclinical model. Furthermore, additional treatment combinations are currently being tested to determine the best approach, including varying the timing/sequencing of therapies and the radiation dose/fractionation.

To further understand the mechanism for decrease in tumor growth resulting from combination XRT and anti-PD-L1 antibody treatment, flow cytometry was used to characterize the tumor immune microenvironment. When analyzing only live cells, there was a higher percentage of CD8+ cytotoxic T cells in the tumors of control mice compared to flank tumors from mice that received systemic anti-PD-L1 antibody treatment and radiation treatment to the leg tumors. However, flow cytometry showed strong differences in activation between the tumor infiltrating lymphocytes in the control group compared to treated mice. Both flank and leg tumors from treated mice had significantly more CD8+ cytotoxic tumor infiltrating T cells expressing PD-1. Additionally, the XRT-treated leg tumors showed a significantly higher percentage of CD8+ cytotoxic T cells expressing CD44, a marker of T cells that are active after antigen presentation. Although the decreased T cell infiltration in treated tumors does not indicate a mechanism for decreased tumor growth in mice treated with radiation and anti-PD-L1 antibodies, the differences in activation can potentially account for these differences. The increased expression of both PD-1 and CD44 suggests that the tumors from mice treated with radiation and anti-PD-L1 are experiencing increased rates of tumor antigen presentation, which could be one mechanism for decreased tumor growth in the treated mice. It is also possible that CD335+ NK cells play a role in the tumor microenvironment, as supported by the flow cytometry data indicating an increase in the mice treated with anti-PD-L1 and XRT. Lastly, the survival advantage is lost when blocking CD8 in the mice, suggesting a key mechanistic role for CD8+ cells in the immune response. Additional mechanistic roles of the immune cells are being investigated, since the immunity triggered by combination immune checkpoint and radiotherapy is complex. [20]

Emerging clinical data indicates about 3% of patients with prostate cancer have a high tumor mutation burden (microsatellite instability-high or mismatch repair deficit) and they are responsive to anti-PD-1/PD-L1 agents, with 45% (5 of 11 patients) experiencing durable clinical benefit [21]. National Comprehensive Cancer Network guidelines for metastatic CRPC include consideration of testing tumor mutation burden and second-line treatment by pembrolizumab. As clinical trials develop to test PD-agents for prostate cancer treatment, it is important to recognize that an immune checkpoint treatment combined with radiotherapy may provide an even greater response rate than monotherapy. The preclinical model presented herein provides a framework for further investigating the optimal approach for combining radiotherapy and PD-agent that can be carried into future clinical trials.

## Conclusions

Using an immune-intact mouse model for the important clinical entity CRPC, survival is dramatically improved by 70–130% when radiotherapy is combined with anti-PD-1 or anti-PD-L1 immune checkpoint inhibitor, respectively, compared to monotherapy. The immuno-radiotherapy treatment response mechanism involves CD8+ cells, suggesting activation of the immune system that is not observed with monotherapy. An abscopal treatment effect was observed for an unirradiated tumor distant from an irradiated one in the same animal, suggesting the potential for the immune system treating widespread metastatic disease. These data provide strong preclinical evidence for a combination treatment approach for CRPC using radiotherapy and immune checkpoint inhibitor, which can inform the design of future clinical trials.

## Additional file

Additional file 1: **Figure S1.** Immunoblot for PD-L1. **Figure S2.** Tumor graft volumes from treatment start until terminal endpoint. **Figure S3.** Flow cytometry gating strategy. **Figure S4.** Flow cytometry for CD4+ tumor infiltrating lymphocytes. (DOCX 521 kb)

## Data Availability

All data generated or analyzed during this study are included in this published article and its Additional file.

## Abbreviations

CRPC: Castration-resistant prostate cancer
IACUC: Institutional Animal Care and Use Committee
iRT: immune checkpoint inhibitor combined with radiotherapy
NK: Natural killer
PD-1: Programmed cell death protein 1
PD-L1: Programmed death-ligand 1
PSA: Prostate-specific antigen
TILs: Tumor infiltrating lymphocytes
XRT: X-ray radiation treatment
