# Supplementary material for: Combination immunotherapy and radiotherapy causes an abscopal treatment response in a mouse model of castration resistant prostate cancer
Source: J Immunother Cancer. 2019 Aug 14;7:218. doi: 10.1186/s40425-019-0704-z (PMC6694548; doi:10.1186/s40425-019-0704-z)
Supplement: Supplementary file 1 — Figure S1. Immunoblot for PD-L1. Figure S2. Tumor graft volumes from treatment start until terminal endpoint. Figure S3. Flow cytometry gating strategy. Figure S4. Flow cytometry for CD4+ tumor infiltrating lymphocytes. (DOCX 521 kb) [file 40425_2019_704_MOESM1_ESM.docx]

**Supplementary Information**

The expression of PD-L1 was found to be dynamically altered by radiation treatment, depending on the radiation dose administered. The highest expression of PD-L1 in Myc-CaP tumor cells was found to be 10 Gy, compared to 0, 2, and 20 Gy treatments (**Supplementary Fig 1**). Thus, this dose was selected for the preclinical tumor graft model.


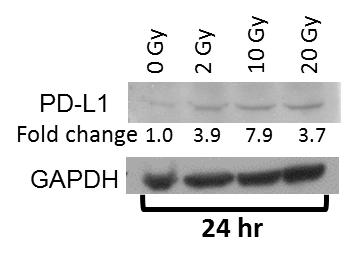


**Supplementary Fig 1.** **Immunoblot for PD-L1.** Myc-CaP cells were treated with radiation at indicated doses and then incubated for 24 hours. Total protein was extracted and probed as indicated. Bands were quantified, corrected for GAPDH expression (ImageJ). Fold change is normalized to 0 Gy (unirradiated) control.

Mice were treated with only 8 days of therapy that included two doses of X-ray radiation treatment combined with either anti-PD-1 or anti-PD-L1 immune checkpoint inhibitor. After the initial treatment responses for both local and distant (unirradiated) tumors during the first 2-3 weeks, the tumors grew and eventually the mice reached the predetermined endpoint. As an expansion of the data shown in **Fig 1A** and **Fig 1B**, the complete tumor growth curves are shown (**Supplementary Fig 2**).


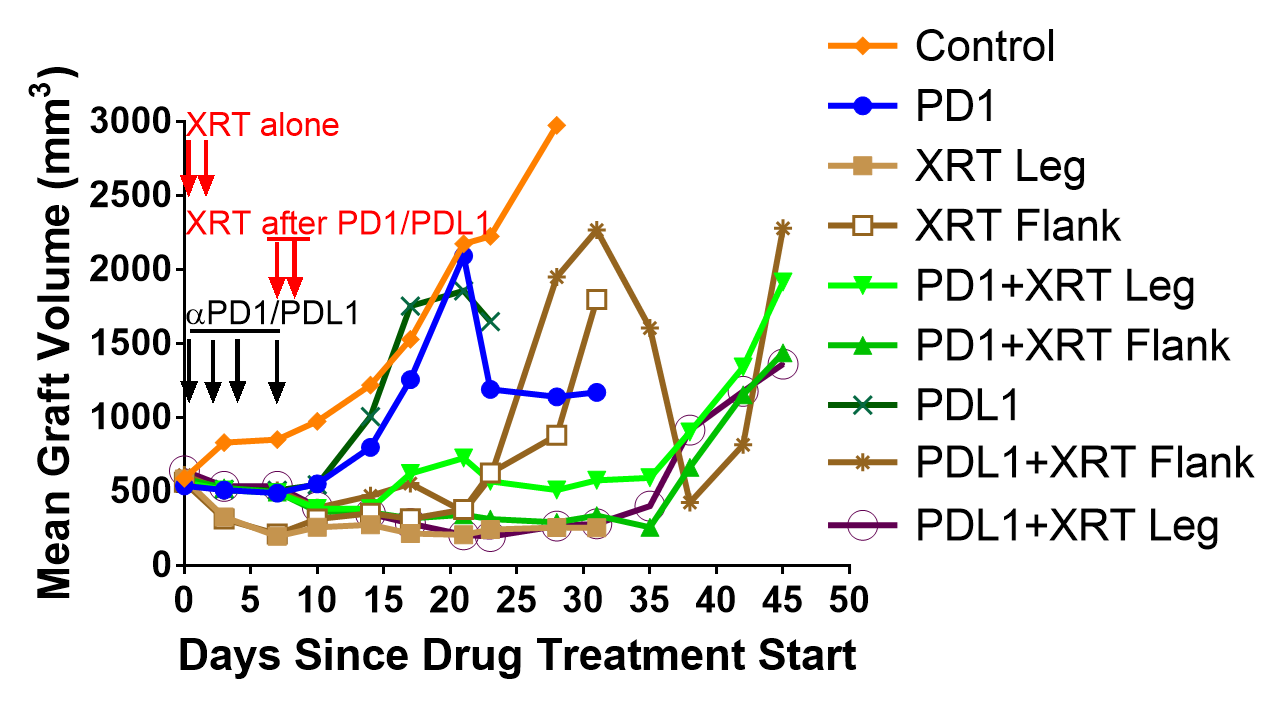


**Supplementary Fig 2. Tumor graft volumes from treatment start until terminal endpoint.** Mice were treated with the indicated therapy and graft volumes were measured 2-3x per week until a predetermined endpoint was reached. Mean growth curves appear more erratic beyond 3 weeks of treatment due to fewer remaining mice in each group. Error bars not shown for figure clarity.


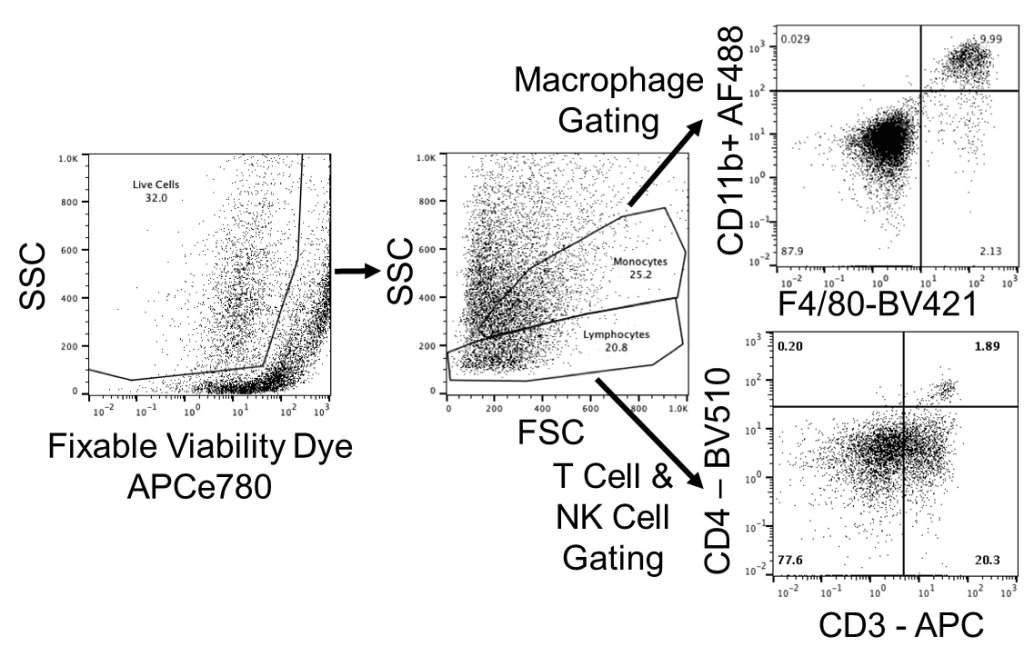


**Supplementary Fig 3. Flow cytometry gating strategy.** Two panels of fluorophores were used to analyze T cell populations and macrophage/NK cell populations. Only live cells were analyzed by the initial gate using the fixable viability dye APCe780. Next, SSC/FSC measures allowed separate gating of the lymphocyte and monocyte populations, with the fluorophore panels testing each staining characteristic.

CD4+ tumor-infiltrating immune cells were characterized by flow cytometry. Although there was no significant change in the total CD4+ cells within tumor grafts, the subset of PD1+ CD4+ was enriched after treatment with anti-PD-L1 antibody in both the irradiated leg graft and unirradiated flank graft in the same mice (**Supplementary Fig 4**).


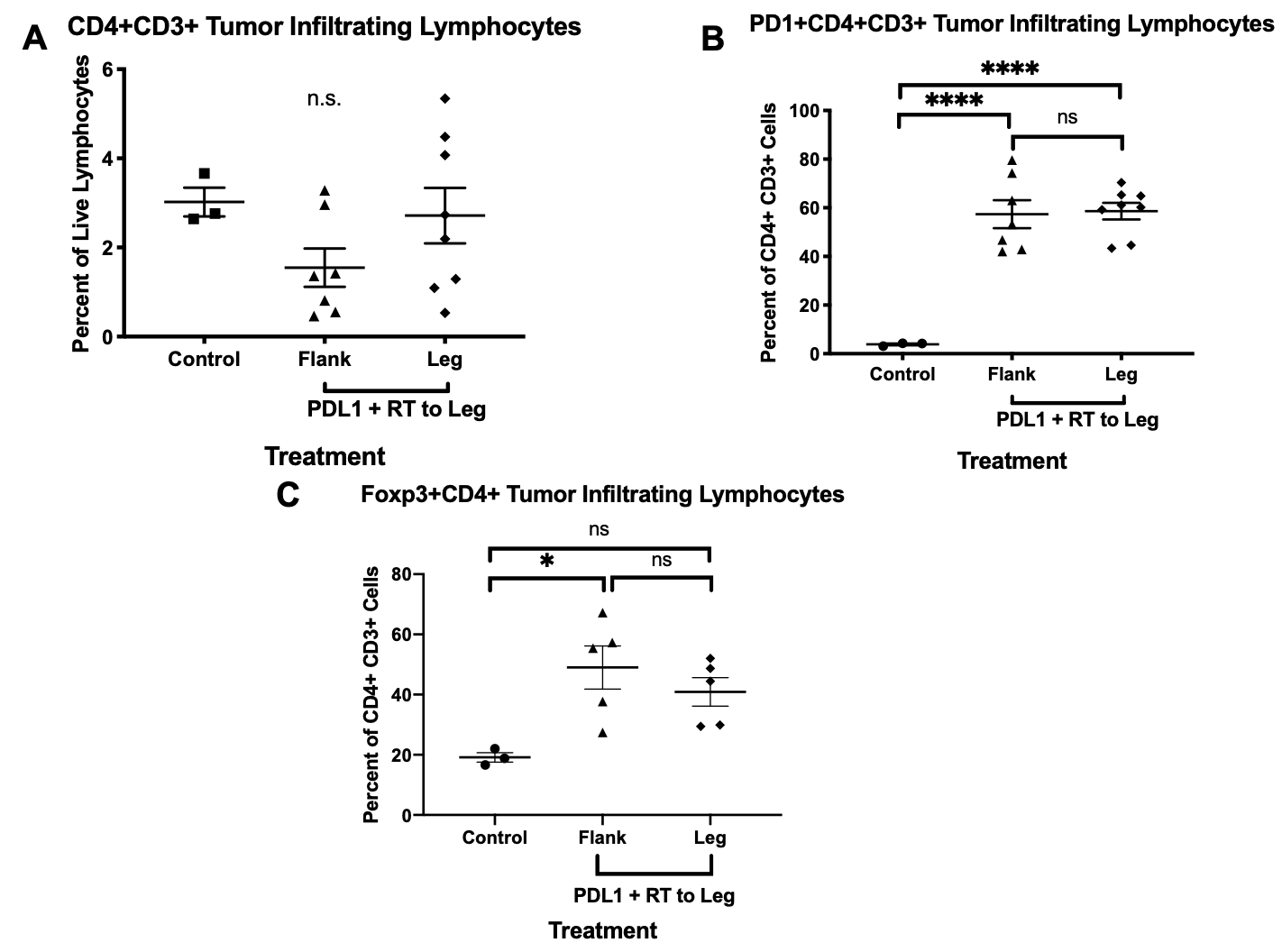


**Supplementary Fig 4. Flow cytometry for CD4+ tumor infiltrating lymphocytes. A and B.** Flow cytometry of CD4+ cells within Myc-CaP grafts in mice with untreated control tumors (N=3), flank tumors from mice treated with anti-PD-L1 and XRT to leg tumor (N=7), or leg tumor that received direct XRT and ant-PD-L1 treatment (N=8). Enrichment in the PD1+ CD4+ tumor infiltrating lymphocytes was observed after treatment with anti-PD-L1 antibody in both the irradiated leg tumor and unirradiated flank tumor in the same animals. Additionally, the T regulatory Foxp3+ CD4 T cells population was enriched in the flank tumors. Error bars represent ± SEM; *****P* < 0.0001, two-way ANOVA test.
